# Case Report: Two highly unusual adrenal tumors presenting with hypertension: a giant cystic pheochromocytoma with an ipsilateral large renal parapelvic cyst and a giant adrenal myelolipoma

**DOI:** 10.3389/fcvm.2025.1541880

**Published:** 2025-03-04

**Authors:** Ali Hakan Konuş, Kader Uğur, Erhan Aygen, Cihat Tektaş, Fatih Durumlu, Muhammet Çalık

**Affiliations:** ^1^ Department of Cardiology, Bingöl State Hospital, Bingöl, Türkiye; ^2^ Department of Endocrinology, Faculty of Medicine, Fırat University, Elazığ, Türkiye; ^3^ Department of General Surgery, Faculty of Medicine, Fırat University, Elazığ, Türkiye; ^4^ Department of Urology, Bingöl State Hospital, Bingöl, Türkiye; ^5^ Department of Radiology, Bingöl State Hospital, Bingöl, Türkiye; ^6^ Department of Pathology, Faculty of Medicine, Fırat University, Elazığ, Türkiye

**Keywords:** giant cystic pheochromocytoma, giant adrenal myelolipoma, adrenal tumor, large renal parapelvic cyst, hypertension, orthostatic dizziness

## Abstract

**Background:**

Giant cystic pheochromocytoma and giant adrenal myelolipoma are two highly uncommon masses. There are difficulties in diagnosis and management of both types of giant (>20 cm) adrenal tumors.

**Case 1:**

A 56-year-old male patient applied with complaints of headache and high blood pressure. A mass was palpated in the left upper quadrant. The average 24-hour ambulatory blood pressure was 146/93 mm Hg. Computed tomography revealed a huge left adrenal cystic mass measuring 22 × 17 cm. A large left renal parapelvic cyst measuring 6 × 5.5 cm was also observed. Urine metanephrine and normetanephrine values were high. The patient was diagnosed with pheochromocytoma. It was decided to remove the adrenal cystic mass and renal parapelvic cyst with open surgery. Severe hypotension occurred during the intraoperative and early postoperative periods, and severe orthostatic dizziness occurred during the in-hospital stay and two months of outpatient follow-up. The patient's urine metanephrine and normetanephrine levels returned to normal. The average 24 h ambulatory blood pressure was 122/69 at six months.

**Case 2:**

A 53-year-old male patient was admitted with complaints of high blood pressure, accompanied by mild headache and dizziness that had been ongoing for several months. A mass was palpated in the right upper quadrant. The average 24 h ambulatory blood pressure was 151/91 mm Hg. Abdominal computed tomography revealed a 24 × 16 × 22 cm solid mass with diffuse fat density originating from the right adrenal gland. Laboratory studies and endocrine investigations were normal. With the diagnosis of adrenal myelolipoma, a mass weighing 4,229 g was surgically removed. The patient was normotensive without medical treatment during the two-year follow-up after the operation.

**Conclusion:**

To our knowledge, our case of giant cystic pheochromocytoma accompanied by ipsilateral large renal parapelvic cyst, which is the first in the literature, reports the management of severe hypotension in the perioperative period and severe orthostatic dizziness in the two-month follow-up. Non-functional adrenal myelolipomas can cause hypertension with mass effect. Our second case is one of the largest adrenal myelolipomas in literature. Although surgical removal of giant masses is difficult, successful surgeries have resulted in resolution of hypertension in our cases at mid-term follow-up.

## Introduction

Adrenal tumors are mostly benign and non-functional. They are often detected incidentally by imaging techniques because they can be clinically asymptomatic or have non-specific symptoms. Giant adrenal tumors are extremely rare and may present clinically more challenging scenarios. We describe two highly unusual cases of giant adrenal tumors, each presenting solely with hypertension (HT), both of which were successfully managed.

## Case 1

A 56-year-old male patient, who had no previous medical history, applied to our hospital's HT clinic with complaints of mild headache and high blood pressure for 10 days. The patient did not complain of any other symptoms, including palpitation, diaphoresis, chest or abdominal pain, and abdominal distension. There was no significant family history. On physical examination, the patient's blood pressure was measured as 150/90 mm of Hg in both arms with a heart rate of 87 bpm. The patient's body mass index was 24.3 kg/m^2^. Cardiac auscultation was normal. A mass was palpated in the left upper quadrant; however, no abdominal asymmetry was observed.

The patient's complete blood count and biochemical values were normal. The average 24 h ambulatory blood pressure (ABP) was 146/93 mm Hg. The electrocardiogram (ECG) showed sinus rhythm with a rate of 79 bpm and no abnormalities. Transthoracic echocardiography (TTE) did not reveal left ventricular hypertrophy. Abdominal ultrasound detected a huge cystic mass in the left upper and lower quadrant of the abdomen. Tests for hormonal activity of the adrenal gland (Table 1) revealed high levels of metanephrine and normetanephrine in urine. The urine metanephrine value was 36,693.2 µg/24 h (reference range: 88–444 µg/24 h), and the urine normetanephrine value was 1,296.3 µg/24 h (reference range: 52–341 µg/24 h). The patient was diagnosed with pheochromocytoma (PHEO). Biochemical tests (calcitonin, parathyroid hormone, and serum calcium) and thyroid gland ultrasonography were normal. Thus, medullary thyroid carcinoma and parathyroid hyperplasia were not detected, and multiple endocrine neoplasia type 2 (MEN-2) was excluded. The adrenocorticotropic hormone (ACTH), dexamethasone suppression test and 24 h urine cortisol levels were normal. The serum dehydroepiandrosterone sulfate, plasma renin activity and aldosterone were also normal.

**Table 1:** Table showing pre-operative and post-operative parameters of patients in case 1 and case 2.

Variables	Case 1		Case 2	
	Pre-operative	Post-operative	Pre-operative	Post-operative
Hemoglobin, g/dl	14.1	13.4	14.8	14.1
Glomerular filtration rate, ml/min/1.73 m^2^	102	98	110	104
ACTH, pg/ml, (RR: 10–60 pg/ml)	48	41	37	–
DST (1 mg), µg/dl	0.85	–	0.92	–
Cortisol (RR: ≤46 µg/dl)	22.2	18.8	24.2	–
Dehydroepiandrosterone sulfate, µg/dl	62.6	–	57.4	–
Testosterone, (RR: 1.96–6.77 µg/L)	3.71	–	3.9	–
Aldosterone, (RR: 7–30 ng/dl)	22.1	24.2	18.5	–
Plasma renin activity, (RR: 0.96–4,18 ng/ml/h)	3.1	3.2	2.9	–
Urine Metanephrine (RR: 88–444 µg/24 h)	36,693.2	78.6	82.3	–
Urine Normetanephrine (RR: 52–341 µg/24 h)	1,296.3	290.2	124.4	–
Cortisol after ACTH stimulation test, µg/dl	–	Basal:18.8 At 1 h: 32.4	–	–

ACTH, adrenocorticotropic hormone; RR, reference range; DST, dexamethasone suppression test.

**Figure 1:**
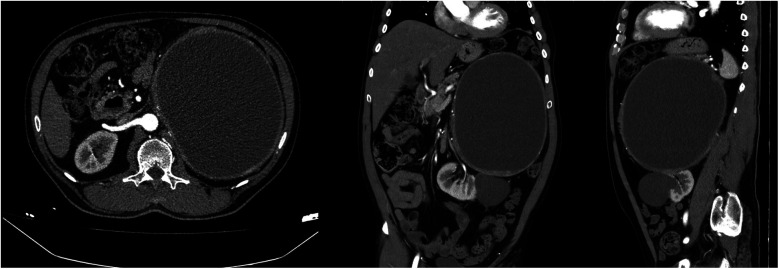
Computed tomography images of a giant left adrenal cystic mass and a left renal large parapelvic cyst.

Contrast-enhanced computed tomography (CT) revealed a huge cystic mass measuring 22 × 17 cm, originating from the left adrenal gland. The mass has regular borders and a contrast enhancement of the wall. Because of this huge mass, the left kidney appeared malrotated, was displaced inferomedially and was observed in the para-aortic area (Figure 1). A large parapelvic cyst measuring 6 × 5.5 cm was also observed in the left kidney (Figure 1). The patient underwent 18 F-fluorodeoxyglucose positron emission tomography (PET)-CT imaging for diagnosis confirmation and metastasis determination and no metastatic involvement was detected.

The decision to remove the left adrenal gland and left renal parapelvic cyst via open surgery was made by the multidisciplinary team. The patient was given doxazosin as an alpha-adrenergic antagonist and propranolol as a beta-adrenergic antagonist in the preoperative period to prevent a hypertensive crisis that may occur during surgery. To prevent hypotension that may develop during and after the surgery, we gave isotonic saline infusion in a controlled manner for two days before the operation. First, the fluid in the cystic mass was aspirated. Then, the rest of the mass and the parapelvic cyst were removed in a two-hour operation. The patient developed acute hypotension after the cystic content was removed without any bleeding during the procedure. Therefore, the patient's blood pressure was controlled with intravenous crystalloid fluid and norepinephrine infusion. The initial norepinephrine dose was 0.1 μg.kg^−1^.min^−1^, and the dose was titrated to maintain mean arterial pressure ≥65 mmHg. During the postoperative follow-up, norepinephrine was gradually reduced and then stopped. Immunohistochemistry revealed chromogranin, synaptophysin and vimentin positivity and S100 negativity, which were consistent with PHEO. Ki-67 index was determined as 3%. Adrenal Gland Scaled Score (PASS) was measured as 10. PET-CT scan did not detect any metastatic focus. The patient was referred for genetic testing to investigate the hereditary PHEO mutation.

**Figure 2:**
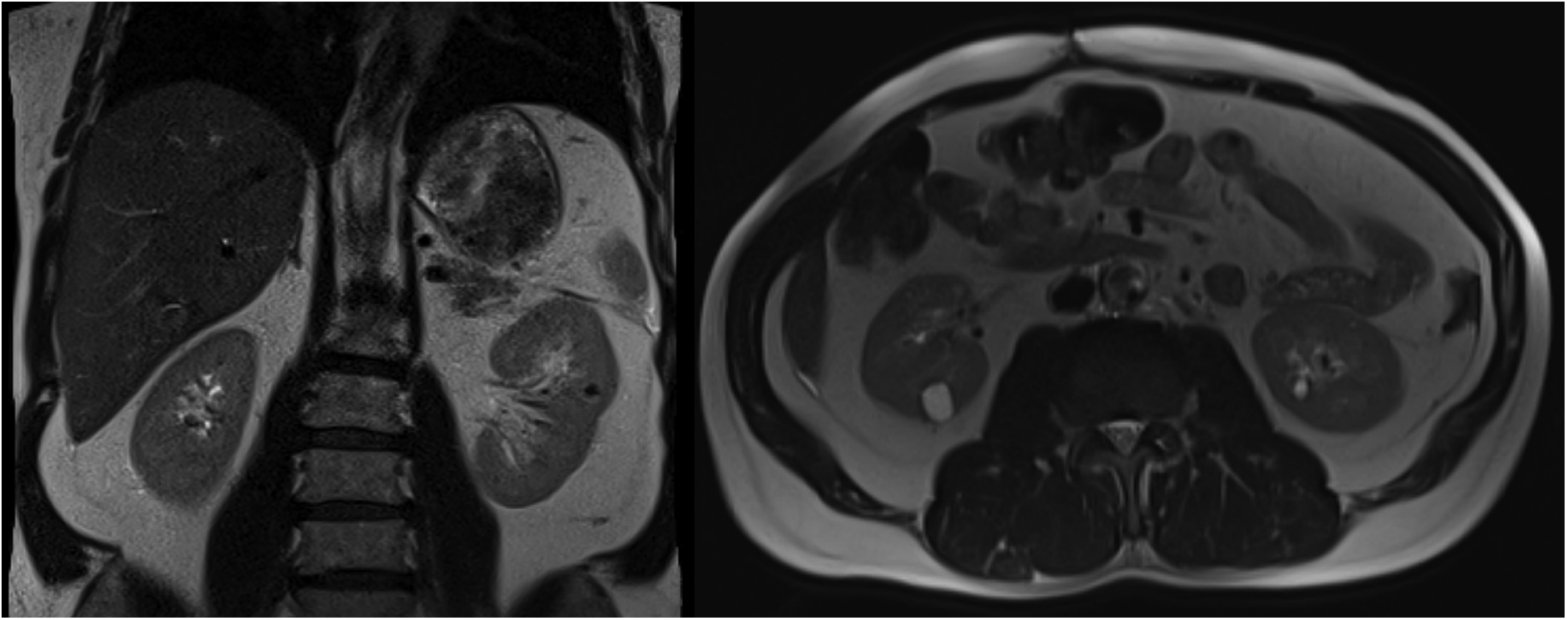
Magnetic resonance images at 1 year postoperatively showing successful resection of a giant cystic pheochromocytoma.

The patient's urine metanephrine and normetanephrine levels returned to normal. The patient complained of dizziness when standing up during postoperative in-hospital follow-up. In the etiological evaluation of orthostatic dizziness, we did not detect anemia, thyroid dysfunction, renal failure, and electrolyte disturbances. There was no dehydration. Adrenal insufficiency was not detected with cortisol levels and ACTH stimulation test (Table 1). Plasma renin activity and aldosterone values were normal (Table 1). Cardiac functions were detected as normal according to ECG and TTE. Tilt test was not performed because it was not tolerable. After discharge, the patient's complaint of severe orthostatic dizziness continued for two months. The average 24-hour ABP was 102/61 mm Hg at the first-month follow-up. Severe orthostatic dizziness was managed with a non-pharmacologic approach, which included increasing salt and fluid intake, modifying dietary habits, isometric exercises, and other lifestyle changes. Despite all this, the patient's mobility was severely restricted. The patient developed deep vein thrombosis in the left lower extremity. Orthostatic dizziness gradually improved after the second month without medical treatment. The average 24 h ABP was 122/69 at six months. The successful result in the control magnetic resonance imaging at one year is shown in Figure 2.

## Case 2

**Figure 3:**
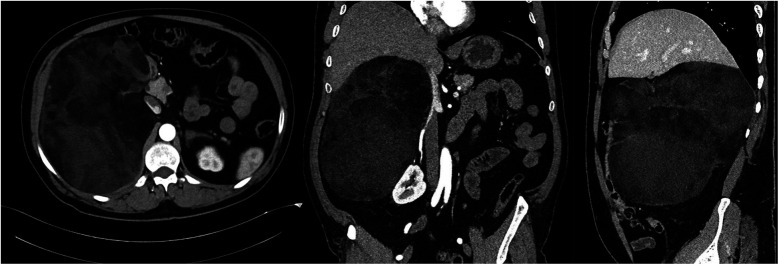
Computed tomography images of a giant right adrenal fatty mass.

A 53-year-old male patient with no previous medical history was admitted to our hospital's HT clinic with complaints of high blood pressure, accompanied by mild headache and dizziness that had been ongoing for several months. The patient had no other symptoms. The clinical examination revealed a class I obesity with a body mass index of 32.8 kg/m^2^, a pulse at 78 bpm and high blood pressure of 160/90 mm of Hg in both arms. The average 24 h ABP was 151/91 mm Hg. ECG showed sinus rhythm with a rate of 72 bpm and no abnormalities. TTE did not show left ventricular hypertrophy. A mass was palpated in the right upper quadrant. Abdominal ultrasound revealed a large hyperechoic mass with hypoechoic areas adjacent to the liver. Laboratory studies were normal. 24 h urine catecholamine levels and the rest of the endocrine investigations were normal (Table 1). Abdominal CT revealed a 24 × 16 × 22 cm solid mass with diffuse fat density originating from the right adrenal gland, starting from the left subdiaphragmatic region, extending to the pelvis, and filling the retroperitoneal area (Figure 3). It was causing compression in the inferior vena cava. The patient was diagnosed with adrenal myelolipoma (AML). The patient underwent open surgery, and an encapsulated mass weighing 4,229 g was removed. Histopathological examination reported that the mass contained a mixture of mature adipose tissue and myeloid tissue.

**Figure 4:**
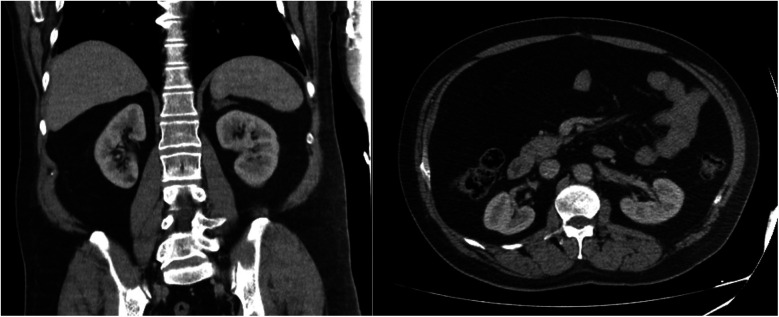
Computed tomography images at two years postoperatively showing successful resection of a giant adrenal myelolipoma.

After discharge, during the two-year follow-up, the patient had no complaints and did not require any anti-hypertensive medication. Abdominal ultrasound performed at 2 months showed no mass. The average 24 h ABP at three months was 127/81. Home blood pressure monitoring remained normal for two years. An abdominal CT scan performed in the second year of follow-up did not reveal any residual mass (Figure 4).

## Discussion

PHEO is a rare neuroendocrine tumor that originates from the chromaffin cells of the adrenal medulla and secretes high levels of catecholamines. PHEO is most common in people between the ages of 30 and 50. PHEO is a difficult tumor to detect and should be evaluated appropriately to avoid adverse outcomes. PHEO is present in approximately 0.01%–0.1% of the hypertensive population (1). However, all PHEOs are most often detected incidentally by an adrenal mass using imaging methods. Our case of giant (>20 cm) functional cystic PHEO presented with HT and was guided to diagnosis by examination. PHEOs are mostly solid neoplasms. Approximately 20% of all PHEOs are cystic (2). Cystic forms must be differentiated from other abdominal cystic masses. While PHEO is rare, the cystic form is even rarer, and giant (>20 cm) cystic PHEO has been reported in a few cases in the literature.

Unlike solid PHEO, cystic form is often asymptomatic and tends to be relatively non-functional. The cystic form tends to be larger, and the size of the mass is considered a predictor of the aggressiveness of the tumor. For this reason, even if they are asymptomatic, cystic forms are thought to have a significant malignancy potential and can be lethal. A PASS score of 4 or higher is used to evaluate the malignancy potential of PHEO (3). In our case, a PASS score of 10 suggests that the malignancy potential of the tumor is high. All cystic forms should be removed surgically to prevent cardiovascular complications due to catecholamine release in functional forms and prevent the development of malignancy in all. Surgery of giant PHEOs is difficult. The main reasons for this are that the tumor size brings many anatomic adjacencies with it, manipulations during surgery can cause a hypertensive crisis, and removal of the functional mass can cause severe hypotension due to the withdrawal of catecholamines from the body.

In our case, the cystic mass had not invaded the surrounding tissues. First, the cyst content was aspirated, then the remaining part of the mass was removed. During the procedure, the patient developed severe hypotension due to withdrawal of catecholamine. Hypotension was managed with intravenous fluids and vasopressor agents. After the early postoperative period, the patient no longer needed vasopressors. However, the patient had severe orthostatic dizziness. Even after discharge, severe orthostatic dizziness continued for two months.

Severe hypotension has been reported during surgery in the literature (4). There is volume contraction associated with excess catecholaminergic activity in PHEO patients. A high sodium diet and fluid intake are recommended to prevent intraoperative and postoperative hypotension in PHEO patients. We gave our patient sodium-containing intravenous fluids as slow infusion for two days in a controlled manner during the preoperative period while he was under antihypertensive treatments. The aim of this treatment was to prevent severe hypotension that may develop after surgical removal of the mass. The preoperative intravenous fluid we administered to our patient may have prevented mortality and morbidity due to severe hypotension that developed during the procedure and facilitated recovery from severe hypotension.

To our knowledge, there have been no reports of mid-term severe orthostatic dizziness in the literature after the removal of functional giant PHEOs. After surgery for PHEO, recovery of sympathetic function to normal is expected to be rapid, and hypotension and orthostatic dizziness are not expected to last long. In our case, other possible causes of hypotension and orthostatic dizziness were excluded by laboratory parameters. Blood loss, cardiac disfunction, adrenal insufficiency, and any other responsible pathology were not observed. Therefore, hypotension and orthostatic dizziness were associated with the withdrawal of catecholamines from the body. This postoperative complication, which causes severe mobility restriction in the patient, should be managed well. In our case, we managed orthostatic dizziness with a non-pharmacologic approach. We aimed primarily to expand blood volume through a high-sodium diet and increased fluid intake both in the hospital and post-discharge period. Dietary habits adjustment and lifestyle changes were also applied for orthostatic dizziness. Although limited mobilization may have caused deep vein thrombosis, severe orthostatic dizziness resolved after two months of follow-up. All these suggest that postoperative adaptation may take a long time after catecholamines are withdrawn from the body.

PHEOs can be sporadic or hereditary. They can also be part of genetic syndromes such as MEN-2, Von Hippel-Lindau disease, and Neurofibromatosis type 1. Our patient's age (54) and large tumor size suggest that our case is probably sporadic. In our case, we confirmed that MEN-2 syndrome was not present with laboratory and imaging. The patient was referred to genetic testing for hereditary PHEO.

Benign renal cysts occurring near the renal pelvis or pedicle are called parapelvic cysts. Parapelvic cysts are rare and mostly asymptomatic. Large (>5 cm) parapelvic cysts may cause compression of the renal pelvis and vessels, leading to hematuria, infection, hydronephrosis, and renovascular HT. Symptomatic or complicated parapelvic cysts require intervention. We did not detect any symptoms or complications related to the parapelvic cyst and excised the parapelvic cyst during open surgery. To our knowledge, our case is the first to report a giant cystic PHEO originating from the adrenal gland accompanied by a large renal parapelvic cyst.

AML is an uncommon benign, mostly non-functional adrenal tumor that is composed of mature adipose tissue and myeloid tissue. AML is generally diagnosed between the ages of 40–70 incidentally (5). It is usually small, and giant AML (>20 cm) is highly rare. The preferred imaging modality for AML diagnosis is CT, which shows the fatty mass. CT is usually sufficient for diagnosis, but magnetic resonance imaging and histopathological examination may be required to differentiate it from lipoma, liposarcoma and renal angiomyolipoma, which contain fat and are non-functional. Surgical removal of symptomatic tumors or myelolipomas larger than 7 cm is recommended (6).

AML is mostly asymptomatic. The most common symptoms are abdominal pain, flank pain and abdominal mass. Some cases may present high blood pressure and associated symptoms. Functional and non-functional forms of AML may be associated with HT. Functional AML may lead to HT through hypersecretion of catecholamines, cortisol, aldosterone and androgens (7–10). Currently, the relationship between non-functional AML and HT has not been clearly elucidated. In a study including 369 AML patients, it was determined that HT normalized in 14 patients in one-year follow-up after surgery (11). It was hypothesized that non-functional AML, especially its large forms, causes HT by compressing the kidneys and/or renal vessels with its mass and fatty tissue content. In our case, complete remission of HT continued for two years after surgery. Our case supports the possibility that such a large fatty non-functional AML causes HT through mass effect. Our case of non-functional AML presenting with HT was diagnosed through the suspicion of an abdominal mass on initial physical examination and is one of the largest AML cases reported in the literature (6, 12). Adrenal myelolipomas are mostly benign and encapsulated, and do not cause adhesion or infiltration to surrounding tissues, even in their giant forms. There are a few cases reported in the literature where the difficult surgery of such large adrenal myelolipomas can be successfully completed with laparotomy, without damaging surrounding tissues and organs. In our case, the removal of the mass and the right adrenal gland was successfully completed.

It was observed that the patient who developed HT due to the mass effect maintained blood pressure normalization after the removal of the lesion during the two-year follow-up. This is an extremely uncommon case showing the mid-term, complication-free success of such huge AML masses.

## Conclusion

Physical examination allowed early diagnosis of two highly uncommon giant adrenal masses in patients with uncomplicated HT at first presentation. Due to cost-effectiveness, diagnostic studies are recommended when conditions requiring investigation of secondary HT occur. However, as in our cases, a mass can be detected by abdominal palpation in patients presenting with uncomplicated HT, and the patient can be referred to imaging methods.

Diagnosis and management of PHEO are difficult. To our knowledge, our case of giant cystic pheochromocytoma accompanied by ipsilateral large renal parapelvic cyst, which is the first in the literature, reports the management of severe hypotension in the perioperative period and severe orthostatic dizziness in the two-month follow-up. Severe orthostatic dizziness due to catecholamine withdrawal may last a long time and patients may need to be monitored for this complication.

Non-functional AML may be responsible for HT with mass effect. Giant forms of AML are quite rare, and our second case is one of the largest AMLs in literature. Despite the difficulty of diagnosis and surgery, our AML case was followed successfully for two years without HT and symptoms after the mass was removed.

## Data Availability

The original contributions presented in the study are included in the article/Supplementary Material, further inquiries can be directed to the corresponding author.
